# Book Reviews

**Published:** 1816-04

**Authors:** 


### CRITICAL ANALYSIS

### *OF RECENT PUBLICATIONS*

IN THE

### DIFFERENT BRANCHES OF PHYSIC, SURGERY, AND MEDICAL PHILOSOPHY.

"Scimus et hanc veniam petimusque damusque vicissem."—Hor.

WHILST the Editors were in doubt whether Mr. Rootsey's* communication was suited to their work, a paper in a contemporary journal decided the question.

The candour with which every performance submitted to our notice is usually treated, has, by some, been condemned, as furnishing less amusement than the coarse humour with which journals of general criticism abound. In answer to

---

* See page 275.

these charges, we can only urge, that the gravity of our language appears to us best suited to the importance of our subjects; and, that, having been all of us early in life somewhat roughly treated, we may feel a degree of sympathy for our writing brethren. A charge is now made against us which we least of all expected, but for which we are ready to express our gratitude, as our utmost wish is to preserve a faithful register of every medical event. As soon, therefore, as we learned that objections were made against the remarks on Dr. Philips' paper, contained in our last number, those objections were instantly conveyed to the gentleman who honoured us with the article, with a request that he would either defend himself, or instruct us all in what manner we might confess our errors with the best grace. We have received the following answer, which we transcribe in his own words:—

GENTLEMEN,

I am fully aware of your instructions, "on all occasions to observe the strictest impartiality, to be careful that every remark shall be couched in gentlemanly terms, and that the true spirit and intent of every work shall be entered into and explained in as few words as are consistent with perspicuity." In all this it has been my endeavour to fulfil your wishes; and, upon perusing the article to which you refer me, I am obliged to admit two of the charges, namely, ignorance of Dr. Philips' intention, and an appearance of sarcasm in some of my expressions. The first, I hope, is venial, if not from the obscurity of the subject, at least from my confession: of the second I was not aware at the time, but, on a re-perusal, am forced to admit that some passages will bear such an implication.

It now becomes me to clear myself from wilful misrepresentation.

The first charge is, that I have taken no notice of Dr. P.'s former paper, though connected with that under consideration.

2dly, That I require respiration should be attended to in whatever relates to the actions of the heart, though the experiments in the last paper are unconnected with that process.

3dly, That my account of another experiment is unintelligible, because I speak of the extremities of the nerves when mentioning experiments on the brain.

The other charges shall be transcribed at length.

As to the first objection, I conceive the paper has been already noticed in your Journal. I, however, made it my business to peruse it, and fancied that in this, as in most of the the French experimental physiology, one great error prevailed in not distinguishing between death and a cessation of action. Without repeating the disgusting cruelties of hot wires applied to the spinal marrow of various incisions, ligatures, &c. and contrasting them with the more tender manner of knocking at head, or suddenly and violently injuring the spinal marrow, these gentlemen should be taught, that, by a slow mode of killing an animal, life may be for some time retained in the various parts, which may, therefore, be affected by stimuli immediately applied to them: but by a violent blow, not only in an animal so easily killed as a rabbit, but even in the vivacious eel, absolute universal death* is often induced, either instantly, in which case the muscles will never contract, that is, stiffening will never take place,—or they will contract so suddenly, that is, stiffening, or, as Dr. P. calls it, a spasm on the muscles, will instantly follow, and be almost as suddenly succeeded by universal death, or a relaxation of the muscles never to be again stimulated. An attention to this single circumstance explains all the difficulties in Haller and in Gallois, without the necessity of any one of Dr. Philips' numerous experiments, or the philosophic candour with which he wishes to treat the French physiologist.

Secondly, in attending to a description of the actions in the heart and arteries of stunned or decapitated animals, the reader can scarcely fail to inquire whether respiration continued in the first, or was artificially maintained in the second.

Thirdly, Such physiologists as still conceive that the nerves originate from the brain, may continue to adopt the term, *origin* of a nerve for its superior extremity; and, in common language, this may be of less consequence, but in philosophical controversy it appeared to me more correct to use the term extremity; and you may perceive by the context that there can be no possibility of doubting which extremity is intended.

You will now give me leave to transcribe from the journal you have done me the honour to send me the following words of my antagonist.

"Mr. Hunter, the reviewer alledges, has anticipated the results of Dr. Philips' experiments. 'The heart's motion,' says Mr. Hunter, 'does not arise from an immediate impulse on the brain, as it does in the voluntary muscles.' This is a peculiarly unfortunate quotation; the result of Dr. Philips' experiments being exactly the reverse of Mr. Hunter's assertion. It appears from them that the heart's motion often does arise from an immediate impulse

* See Hunter on the Recovery of Persons apparently drowned.

on on the brain. No man has a greater respect, I may say veneration, for Mr. Hunter, than the writer of this paper; but I feel no hesitation in affirming, that the works of this great physiologist contain no anticipation of the views afforded by Dr. Philips' experiments."

This respect and veneration for Mr. Hunter may be very great, but I trust some of your readers will think it much lessened when it is more than hinted that so respectable, so venerable a name, is to be eclipsed by Dr. Philips'. Let us examine, then, the passage, and see whether the compliment I wished to pay Dr. Philips in confirming his accuracy by the authority of Mr. Hunter is not well founded.

Mr. Hunter says, "the heart's motion does not arise [*or originate*] from any immediate impulse on the brain." Dr. Philips shows "that the power of the heart is independant of the nervous system." That its ordinary motion may be accelerated by stimuli applied to the brain, or to any part of the body, did not require an experiment for its proof. This will be further explained in my remarks on the following extract.

The words of my antagonist are—

"He then observes, 'Some remarks follow on the effect of communicating sensation,'—motion, he should have said,—' by the nervous ganglia. In these there is nothing new.' The points on this subject, ascertained by the experience of Dr. Philips, are, that the heart obeys stimuli applied to every part of the brain and spinal marrow; and, consequently, that nerves issuing from ganglia, the only nerves which the heart receives, convey the influence of every part of these organs, while those parts of the body not supplied with nerves from ganglia obey only the minute parts of the nervous system from which their nerves arise. Now, either the reviewer knows of some work not known to the public, in which Dr. Philips has been anticipated in this discovery, or he has here stated what he cannot confirm."

I am much obliged to this gentleman for setting me right in substituting the word motion for sensation: perhaps we are both wrong, and, instead of altering the noun, it might be better to alter the verb, using *exciting* instead of *communicating.* It will then be seen, that, as the motion could only follow the sensation, it is of less consequence which term is used. It may serve, however, to puzzle, if that were necessary; but to me the remainder of the paragraph is puzzling enough of itself.

You will not, I am sure, accuse me of inattention to the events passing in the medical world, nor, I trust, doubt the truth of my assertion, that Dr. P.'s paper occupied more of my time and application than all the other works which arrived by by the same packet. If his experiments (I am not speaking of his inferences) really show, or even imply, that the distribution of nerves in the heart is different from that in the rest of the body, or even if they showed any thing in this respect different from what was generally known, I confess it escaped, and still does escape, my notice. If they only showed that the heart sympathizes with every part of the brain, without the necessity of the will, whilst the involuntary muscles only sympathize with the brain according to the directions of the will,—all this, I conceive, is contained in Mr. Hunter's remark, to which I referred your readers, "that the motion of the heart, that is, its regular alternate contraction and relaxation, does not arise from any impulse on the brain, like the voluntary actions of the muscles usually termed voluntary."

After very long study, however, I am somewhat doubtful whether Dr. Philips and his encomiast may not mean more than occurred to me before. The heart is supplied with nerves only from the ganglia, and is affected by stimuli applied to every part of the brain and spinal marrow: *ergo*, nerves issuing from ganglia convey the influence of every part of the brain and spinal marrow; while those parts whose nerves do not issue from ganglia are only affected by stimuli applied to parts of the brain and spinal marrow immediately contiguous to that part of the brain or spinal marrow from which they issue. If this is his meaning, more experiments are required. The muscles of the abdomen, and the intercostal muscles, receive their nerves from ganglia. Are these also affected by stimuli applied to every part of the brain and spinal marrow?

Such, gentlemen, is my defence. I will not, however, assert that Dr. P.'s experiments throw no light on pathology. They may lead us to some important conclusions concerning convulsions, which may be improved by those who draw proper inferences. I sincerely hope, therefore, that Dr. P. will continue his experiments, with less cruelty; and in future I shall relieve myself from too close an attention to his inferences, conceiving it enough to admit the faithfulness of his reports. I have the honour to be,

Gentlemen, &c.

---

*Medico-Chirurgical Transactions, published by the Medical and Chirurgical Society of London.* Vol. VI. 8vo. Longman and Co. London.

THE first article in these Transactions in a long and very valuable paper from Dr. Calvert, giving an account of the origin and progress of the Plague at Malta. On this, as a subject a subject connected with the doctrine of contagion, we shall give as copious extracts, and with as many remarks, as our limits will permit. The paper begins in a manner which arrests our particular attention to these subjects.

"The following communication has two objects in view: first, to give a faithful narrative of the introduction and progress of the plague at Malta, in the year 1813; and, secondly, to ascertain, from induction of facts, the laws of pestilential contagion, so as to direct us in the employment of preservative means; but particularly as relates to the construction of lazarets, and to the admission of people known to be infected within our ports.

"Towards the accomplishment of these two ends, the most prominent circumstances that occurred during the pestilential season are selected, while the principal proclamations and other public documents are given without comment, that the facts themselves may be seen without the colouring which they might receive from argument."

"I do not believe, (concludes Dr. C. in this introductory part,) as it will be seen, that the plague found its way into the island and spread itself from want of exertion on the part of government, or of the department of health; for almost every human means were put in force in conformity with the popular doctrine of pestilential contagion; but the grand and fundamental error, I believe, was wholly and solely in the doctrine itself."

The rest of the paper occupies sixty pages: we can, therefore, only admit an abstract of the historical part.

The following is the history of the vessel and crew which are supposed to have introduced the pestilential contagion into Malta:

"On the 29th of March, a vessel called the San Niccolo arrived at Malta, from Alexandria in Egypt, the master of which informed the officers of health, as he came into port, that he believed the ship was infected with plague, having lost two men during the voyage of what he strongly suspected to have been that disorder, particularly as it was raging at Alexandria when he left that place. One of the men, he said, had a black tumour upon his neck, to which he himself had applied poultices. He also added, that as soon as the two men died, he immediately suspected the nature of their disease; and, by way of precaution, ordered the hatches of the ship to be closed, and kept the men on deck. This happened about a week previous to his arrival in Malta, and during the interval they had eaten nothing but a few biscuits that happened to be left on deck.

"The master and surviving part of the crew, being apparently healthy, were permitted to disembark in the lazaret; not, however, before they had taken the usual precautions of shaving their heads, washing themselves with sea-water, and afterwards with vinegar, and of leaving their clothes behind them in the ship.

"As

"As the crew consisted of men of different nations, they were divided into companies accordingly, each company being provided with two apartments in the lazaret; and, as the captain and his servant were both Maltese, they lived together.

"The whole continued, in appearance, to enjoy the most perfect health till the 1st of April, when, on the afternoon of that day, the captain, while playing at ball, was suddenly seized with head-ach, giddiness, and other symptoms of plague; and he died in the course of about thirty-six hours. His servant, who had also assisted the sick men on board, was seized about the same time with similar symptoms, and he died after a like interval. They were both buried in the lazaret.

"While these things occurred on shore, the usual precautions with regard to the ship were not neglected. She had remained in the middle of the quarantine harbour from the time of her arrival, with two guard-boats stationed near her, to prevent every kind of communication; and she continued in this situation near a fortnight, at the end of which time a number of men were hired, for a considerable sum, to conduct her back to Alexandria.

"The ship and the whole of these men arrived safe in Alexandria, and the cargo was afterwards taken out without a single individual being infected, as appears by the following letters from the British consul at that place, addressed to Lieutenant-general Oaks, the King's commissioner at Malta.

(Translation, No. 1.)

"'May it please your Excellency,

"'It is with the greatest satisfaction I have the honour to inform you of the safe arrival here, on the 4th of May, of the brigantine, S. Niccolo, Captain Alexander Scarneo. Besides, the crew are all in perfect health.

"'As no quarantine is observed at this place, the crew had permission to leave the vessel whenever they pleased. As to the disposal of the cargo, we are in daily expectation of an order from his Highness the Viceroy.

*May 8th*, 1813.'

(No. 2.)

"'In addition to what I had the honour to communicate to your Excellency on the 8th of May, by his Majesty's sloop Badger, respecting the brigantine S. Niccolo, commanded by Alexander Scarneo, I have now to inform your Excellency, that the brigantine has been entirely unloaded, and that the clothing, bedding, &c. have been disembarked; and that I ordered the vessel to be ventilated, washed, fumigated, and white-washed throughout every part, to be painted without, and the sails and rigging to be washed, and the seams pitched. I have the pleasure to add, that no person employed in unloading the brigantine has been attacked with plague, and that this disease has almost entirely disappeared here. (Signed) STEFANO MALTAS, British Consul.

*June 1st*, 1813.'"

We here take our leave of the vessel, cargo, and crew, and return to the island of Malta.

"As the survivors of the original crew continued healthy in the lazaret of Malta, and as the dreaded ship no longer remained in the harbour, the deluded inhabitants began to congratulate themselves on their supposed happy escape.

"But, on the 19th of April, a Maltese physician, Dr. Gravagna, being called to visit a child of the name of Borg in *Strada S. Paolo,* found it in a dying state, of what he then believed to be a typhus fever. He observed a carbuncle on its breast; but, as this was small, and as the family were subject to cutaneous disorders, the real nature of the disease was not suspected. The child had been ill five or six days.

"On the 1st of May, the same physician was again called to see the mother of this child, whom he found affected with fever, accompanied by a painful tumour in the superior inguinal glands. On the 3d, she was delivered of a child of seven months, which died as soon as it came into the world. In the course of the same day, another tumour of an inflammatory nature was perceived in the glands of the other groin of the mother, and she died before the next morning.

"During the sickness of the mother, another child was attacked with fever, which, however, did not prove mortal.

"The father of this unfortunate family, Salvator Borg, had not long to bewail the loss of his wife and infant, before he himself was threatened with a similar fate. On the morning of the 4th, he was attacked with fever, accompanied with glandular swellings in the axilla and groin.

"Dr. Gravagna, being now no longer in doubt as to the nature of the disease, related every thing that had happened to the deputation of health. On hearing the account, they immediately ordered that not only Borg's family, but every individual proved to have had the least communication with it, should be instantly removed to the lazaret; and this order was executed with the greatest care and industry."

Without pursuing the disease further, it is sufficient to remark, that the general opinion at Malta was, that the plague could only be communicated by contact; but, in the instance quoted, not only no contact could be proved, but none was within the reach of probability. Innumerable other instances are produced in which the plague spread in a similar manner, and some in which the closest contact was unattended with any ill consequences. The proclamations, and their strict observance, are next transcribed, with satisfactory remarks: the history then continues—

"In spite of these and many other rules and regulations, the disease continued to spread itself in every part of the city, attacking principally the poor, and those inhabiting small and dirty houses. The veteran soldiers, too, who were placed at the doors of the infected houses, were frequently attacked. On the 18th, there were seven people attacked. On the 19th, three attacks and eight deaths; and on the 20th, eleven attacks and ten deaths, according to the reports."

Whilst the gentleman to whose review we submitted the above article was preparing his remarks, we were favoured with the following communication, of which he has availed himself.

GENTLEMEN,

Having been honoured with a perusal in MS. of Dr. Calvert's Account of the Plague at Malta, I took the opportunity of introducing that gentleman, with his paper, to some persons high in office in this kingdom. Having also considered the subject very maturely, my opinions in writing were submitted to the same authorities. If they will be at all useful, or are thought worthy of your notice, they are quite at your service. I am, Gentlemen, &c.

JOSEPH ADAMS.

***Reasons for doubting the Conclusions drawn by Dr. Calvert from the facts he observed during the Plague at Malta;* by Dr. ADAMS.**

I CAN have no intention to question the accuracy of Dr. Calvert on any incident related from his own knowledge, or even which he found well authenticated during his residence at Malta; but it should be remarked, that when the vessel which is supposed to have introduced the plague arrived at that island, the doctor was in Sicily. In the history of this vessel we are informed—

That two of the crew died during the voyage of a disease which the captain suspected to be the plague.

That, in consequence, he kept himself at a careful distance from such of the crew as he suspected; and gave information, on his arrival at Malta, of all that had happened.

That, on his arrival, he was ordered to the quarantine island in the harbour, where he died of the plague.

That the quarantine was observed with as much severity as possible, and, there is no reason to doubt, with equal fidelity.

That the first subjects who were attacked were in a part of the town distant from the sea side, and in a very close and dirty street.

That the greater part of that family died.

That every precaution was taken to prevent intercourse, and the disease for a time seemed to cease, which was, of course, imputed to the cautions used.

That subsequently the disease appearing in various parts of the town, but never spreading, excepting in the narrow and crowded streets, it was thought advisable to divide the town into districts,

 by by barriers, and to place a sentry at each barrier, in order to prevent any dangerous communication between the different parts.

That, notwithstanding all these precautions, the plague broke out at various parts of the town, and whenever it began in a close or crowded street, it continued to spread.

That, in Dr. Calvert's opinion, it was always conveyed to every new place by the arrival of a person with the disease on him, or seized with it soon after his arrival.

That, on the approach of winter, or on some change of the atmosphere, the disease ceased.

That the ship returned with a fresh crew to the port from which it was supposed to have brought the plague, and, without any quarantine, the crew, which had not suffered in the supposed infected vessel, were permitted to land all their cargo, which they did without injury to the inhabitants.

That almost all who were sent to the city lazaretto died.

That few died who were received into the military hospital; and that the disease did not spread there.

That persons affected with other diseases soon found them converted into the plague.

That one woman among the military, much addicted to drunkenness, was seized with the plague, and died; but that the disease did not spread among the soldiers.

That very few escaped of those who were sent to the civil pest house.

From the above history I should venture to draw the following conclusions:—

That if the men who died during the voyage had the plague, which is highly probable, there is no proof that they brought it from the place at which they received the clean bills.

If they really introduced the plague into Malta, neither certificates nor quarantines are any security; and it is certain that they returned from a port [Malta] which could not give them a clean certificate, to a port free from the plague, without any quarantine, and without introducing the plague.

If the men had the plague during the voyage, it is not more remarkable than that part of the crew of a ship should be seized with mumps during a voyage from a port at which the disease did not exist. This is by no means uncommon; but a much more frequent event is that a crew leaving a port in health shall be seized with influenza during their voyage. Thevenot, in his *Voyage au Levant*, gives a short history of an epidemic catarrh which reigned in Grand Cairo, with such severity, and so universally, that he does not scruple to consider it *si contagieuse que se gagnoit facilement par la communication d'haleine.* The disease, he adds, extended itself so far, that afterwards, when they were at Jerusalem, and at other places round about, it was found those places were afflicted at the same time, and even the corsairs who took them had it at that time. Here a disease, the cause of which evidently only existed in the atmosphere, was pronounced contagious, merely on account of its universality universality in crowded towns and in a ship's crew. The arguments in favour of contagion in influenza are as strong as those concerning the plague. Indeed the plague of Athens, as it is called, is the only contagious disease mentioned by the ancients, if we except Aretæus's suspicions concerning Elephantiasis; yet Thucydides, who gives this character to the disease at Athens, describes it, in its beginning, precisely as an influenza. In its progress it assumed all the forms of a garrison fever., with hospital gangrene.

From this I should conclude that the same constitution of the air as induced the plague in Malta, first introduced it into a crowded vessel; that in the latter the common men only were infected, because they only were crowded. But, that on their arrival at the lazaretto, the captain, having now changed the deck of his ship, on which he was exposed to a constant change of atmosphere, for an island within a confined harbour, so low as only just to rise above the water, was constantly breathing an unchanged atmosphere; and that the great and sudden change from a free atmosphere to the confined air in a flat island, in a good harbour, was sufficient to account for his greater susceptibility; and such is usually the case with fresh comers, who are the first seized with yellow fever.

No one disputes that the quarantine was strictly observed; yet the plague first appeared at a distant part of the town, in a crowded neighbourhood, and a close street. The customary precautions were taken, and, it was supposed, with success; yet the plague afterwards appeared in various parts, in all of which it was supposed to be brought by different persons from the infected parts. But why are we to look for such a cause, when we find that the first persons affected in the town could have had no intercourse with the harbour.

Those who were sick of other complaints found their diseases changed into the reigning epidemic, an observation as old as Thucydides, and constantly occurring with every severe influenza. The question should, therefore, have been, whether these people, among the wealthier or better-regulated communities, communicated the disease? There is not a single proof that they did; and we are expressly told that the female drunkard who was affected in the barracks never infected any other person.

That the persons employed to bury the dead on these occasions suffered, contrary to what happened in European houses, is easily explained. At Malta they fetched the dead from pestilential quarters of the town. Among the Europeans they are taken from houses in which solitary cases have occurred in healthy families.

All these questions are unnoticed by Dr. Calvert, as well as in the progress of the plague at Aleppo, as described by Dr. Russel; yet these two accounts may be considered the only histories of the infectious march of a plague, containing matter sufficient to admit of satisfactory reasoning. They both induce me to believe that the plague, like the yellow fever in the crowded towns of hot climates, like like the influenza in all climates, is a disease arising from a certain constitution of the atmosphere, requiring, besides the said constitution, a situation in which the same atmosphere may remain unchanged for some time, or such a state of the human constitution as renders it particularly susceptible of disease, which is soon converted into the reigning epidemic. Consequently, that the means used for checking the infection are not entitled to any credit, inasmuch as the disease spread during all these cautions in crowded neighbourhoods, and existed, without spreading, in airy and well-regulated communities; and ceased, according to the usual progress of the disease, with a change of temperature.

On one remark I must beg to be a little prolix. Dr. Calvert says, that the disease was traced from one city or one village to another, rarely, as far as my memory extends, revisiting the same district, though sometimes sporadically a little before or after it became completely epidemic. In all these cases he says there was evidence that it was conveyed by a diseased subject, or by a person who came from an infected district.

I know not how to reconcile this account with the cautions which were used, but by supposing that when the disease became epidemic in a town or village, it was afterwards discovered that it first appeared on a new comer, who probably had been, at some former period, in some infected district; but who probably was only the first seized by being more susceptible from the new kind of atmosphere which he breathed. This is not peculiar to epidemics arising from a mere altered constitution in the atmosphere; but occurs even in the contagious. The small-pox commonly attacks Lascars on their arrival in Wapping. No one will suppose that they have introduced it; but they are more susceptible of the slightest impression from a cause to which they are less familiarized than the natives of the port at which they arrive. In epidemics purely atmospherical, and exasperated by the crowding together of the inhabitants, it is much more remarkable. The suspicion of the importation of the yellow fever arose from the fresh-arrived sailors being usually first attacked; but the migration of the disease from one district to another is so exactly similar to another known to be purely atmospherical, that I cannot avoid copying the following account from the late Dr. Heberden's History of the Influenza of 1782, published in the Transactions of the London College of Physicians.

" After three or four days from its first appearance had elapsed, it was observed, in several instances, that when any individual in a family was attacked with this distemper, the greater part of that family, and sometimes the whole, was very soon after seized with it; and that those who were thus seized were not successively but almost all at once taken ill; and frequently with symptoms similar in kind, but differing in degree. In other instances the disease went successively through families; while to others, and those numerous, it was so favourable as only to attack very few in each. It was also remarked, that those whom business or inclination carried carried into the air, and who exposed themselves to the vicissitudes of the weather, were not more subject to the influenza than those whom occupation, accident, or previous indisposition of another kind had confined at home. Very early in June, three families, consisting of seventeen persons, came on the same day to the hotel in the Adelphi buildings: they were all in perfect health when they arrived; and they were all affected the next day with the symptoms of the illness then reigning in London.

"The disease appeared earlier in towns, than it did in the surrounding villages; and in villages earlier than in the detached houses in the neighbourhood.

"In some instances it was observed that the influenza did not shew itself in certain places until some one or more arrived at those places either actually labouring under the disease, or coming immediately from other places, whose inhabitants had been affected by it for some days; while, in other instances, very attentive and intelligent observers could not trace any communication between the families first attacked in the towns in which they resided, and other places, where the disease had previously appeared."

"We have credible information, that a family which came in the Leeward Island fleet, that arrived in England the latter end of September, 1782, from the West Indies (where the disease had not made its appearance), was attacked by it in London in the beginning of October," [yet the disease had ceased in London at least two months; but the new-comers, from their greater susceptibility, were affected. So it was with the English army in Egypt: the new arrived troops were affected before the plague was suspected in the country; and others, who arrived after it had ceased, were affected also.]—See vol. iii. page 54 (art. viii).

Thus, whilst I perfectly agree with Dr. Calvert that the disease is conveyed by the atmosphere, I conceive, contrary to his implication, that a diseased subject is not necessary; but that the constitution of the atmosphere, when that atmosphere is confined, is sufficient to induce the disease in such as are susceptible of it; that, without such a constitution of the atmosphere, it cannot invade any one; and that this atmosphere is more dangerous in proportion as human subjects are more crowded; and that the susceptibility is increased by previous illness, or any debilitating cause.

The practical inferences from the above are—

That, whatever may be the source of the plague, or the cause of its spreading, quarantines have hitherto proved useless. That, should it be thought necessary, on account of the general prejudices in their favour, to continue them, it is but reasonable to keep the crew on board their own ship, where they cannot suffer by landing in a lazaretto dangerously situated, and where they may remain till they are all convalescent, and be allowed to land, after being washed on deck, and cloathed in raiment sent them from shore.

That, as no means were sufficient to prevent the march of the plague from one district to another, or, as it is called, the spreading of the infection during the autumnal months, the business of the police should be to lessen the ravages of the plague during those months, without expecting its extinction till before the change of season. Instead, therefore, of confining every one to pestilential houses and districts, no one should be allowed to enter them; and proper encampments should be prepared for all those who chuse to leave them, which they should be encouraged to do by bringing their sick with them.

If the account given of the fever of Gibraltar in the last volume of the Medico-Chirurgical Transactions is examined, it will be found, in its infectious march, in many respects similar to that of the plague in Malta; and its introduction by a diseased subject, though by no means satisfactory, rests on better grounds than the introduction of the plague at Malta by the suspected vessel.

---

*Institution for administering Medical Aid to the Sick Poor, and assisting them and their Families with the Necessaries of Life during Sickness; and for preventing the Spreading of Contagious Diseases.* 8vo. Dublin, 1815. pp. 27.

Though this performance is intended only to assist the charitable designs of those who drew it up, it is well worth preserving as a medico-statistical record. As a specimen we have selected the following:

"Among the complaints peculiar to women, that come daily under our care, there are scarcely any (leucorrhœa and obstructed menses excepted) for which we are so repeatedly consulted, as for uterine hæmorrhagy. The treatment of this sickness is well understood, and commonly efficacious. But unluckily it sometimes takes place at and even after the critical period, when it is, in most instances, the precursor of or the attendant on uterine cancer. Though a prophylactic regimen may be occasionally serviceable against the accession of this deplorable malady, yet no established cure has been hitherto discovered for it in its confirmed state. In a conversation with Doctor Osiander, junior, on this subject, I heard him advise the extirpation of the diseased part, as is done when a similar evil affects the breast at the same stages of life. It is reported in some medical journals that this operation was performed in Gottingen by Doctor Osiander, senior; and that it has been lately practised by Mr. Dupytrien of Paris. Though I have not seen any historical detail in confirmation of the cases in question, I do not pretend to controvert this assertion of facts on the part of such respectable authorities. But at the same time I hesitate not to maintain that their example will not be often followed. The remedy seems to me to be, in general, equally desperate as the malady which it is intended to remove. Every practitioner knows, that when he is consulted about this complaint, and that he ascertains the existence either of scirrhus or cancer, the disease has, in most instances, already made much progress, occupying pying the entire cervix, with a considerable portion of the body of the uterus, and the whole subjacent region of the vagina, as far anteriorly as the urethra and posteriorly near to or close on the rectum; and that no efficient amputation could be practicable without wounding both or either of these canals, especially the former. We have besides to apprehend the hæmorrhage and other dangerous consequences that must result from so extensive an organic destruction in the pelvis. I would wish to learn how long the persons operated on by the above-mentioned gentlemen survived; or, if they be still in existence, what degree of health do they enjoy. I am inclined to conjecture that each of them laboured under no more than an incipient state of scirrhus, of little extent, or a partial and small induration of either side of the os tincæ, such as the modesty and negligence of our patients seldom permit us to take timely notice of. Even under these less unfavourable circumstances, how can the surgical instrument be employed, without offering violence to the uterus, and dragging it down from its natural situation?

"Many of the complaints that afflict children, being equally common to adults, are noted above. I make no remarks at present on their dentition and its concomitant train of affections; nor on their vomitings, intestinal pains, convulsions, water on the brain, or rickets; all of which are of familiar occurrence in our walks. They are subject to worm fevers, with mid-day and evening exacerbations, which correspond to what some physicians denominate the remittent fever of infants. These are, for the most part, advantageously treated by the administration, on alternate days, of a few grains of rhubarb or jalap with a little submuriate of mercury.

"With respect to general cathartics for infants, the last-mentioned articles are often used: oleum ricini evacuates their bowels as satisfactorily as any matter that can be given to them, though its administration is by times attended with difficulty. Syrup of pale roses and syrup of violets are mild purgatives, particularly suitable to the early stage of infancy. Subcarbonate of magnesia is a corrector of their flatulencies.

"Scrofula, which is far more common in children and young persons than in people of advanced years, and more frequent and obstinate among the poor than the rich, is not very high on our list: nor can I say that its annual number is proportionably greater in Dublin than in most other cities. Many of the means that contribute to obviate, alleviate, or cure this ailment, viz. cleanliness, good air, nutritive diet, warm clothing, and sea-bathing, are in general not within the reach of the poor. Among the numerous tonic remedies recommended in scrofulous cases, cinchona is that of the benefit of which I have the most experience. I often prescribe a little of it to be taken in a state of mixture with a few grains of subcarbonate of soda, and sometimes by itself; and I occasionally interpose a mild purgative. The minor glandular tumours of this malady are at times discussed by their being kept covered with the emplastrum saponis. The topical employment of bruised sorrel leaves (rumex acetosa) are recommended as contributing to the cicatrization of indolent scrofulous ulcers; I tried them on one or two patients with good effect. I have, on many occasions, had cause to be much satisfied with the application of the unguentum nitratis hydrargiri to these, as well as to herpetic sores. I have reason to think that muriate of lime is not employed as often as it merits. It is scarcely to be met with in the shops, though it can be easily made, or an abundance of it may be procured at little or no expense in the manufactories of mild and caustic ammonia.—The French physicians give bark, ferruginous preparations, and muriate of barytes, sometimes, but much seldomer than is done in Great Britain. They are in the daily habit of ordering one or other of the following antiscrofulous medicines, namely, bitter tinctures, such as one obtained from equal parts of gentian and rocket* roots, or that prepared from gentian and rhubarb, with the addition of carbonate of soda; strong decoctions of hops, and of the woody night-shade.† The two last matters are also had recourse to in herpes."

On a future occasion we shall endeavour to compress the nature of this institution in our Intelligence: with this view we are employed in gaining further documents, which we find the more necessary, on account of the different state of society and police in the two capitals.

---

*Dissert. inauguralis medico physiologica sistens historiam Veneni Upas antiar, nec non Experimenta, et ratiocinia quædam de effectibus illius, auctore Schnel. Tubing.* 1815.

The author relates, in the preface, his having been present at the experiments mentioned in the dissertation, and performing the greater part, when he resided at Bern, where they were made, by Prof. Emmert, assisted by his *Prosector* Dr. Meyer; and that the former entrusted the same to him, to form the *material* for his inaugural dissertation. He next touches upon the history of the Upas poison, which, with the exception of Leschenault's account, had been disfigured by fables. The poison employed in the said experiments Prof. Emmert received from Mr. Leschenault: it was exclusively Upas antiar, the same sort which Leschenault and Brodie had already employed in a few preceding experiments.

From the experiments made upon animals with this poison, the following results are deduced:

The Upas antiar is a deadly poison to all animals, both of

* Brassica Eruca. † Solanum Dulcamara.

warm warm and cold blood, though less dangerous to the latter. Its poisonous effects also proceeds from the medulla spinalis, to which the poison must be communicated by means of the blood-vessel system. The symptoms of poisoning are the following:—the respiration and pulse becomes quicker; weakness of the voluntary muscles, and tremor of the limbs, succeed soon after; the animal drops down, vomits, has evacuations by stool and urine, pants for breath with a distended mouth; convulsions and opisthotonus follow; the pulse grows weak and intermittent, is entirely wanting, or scarcely to be felt. After death, the heart is dilated with blood. When the poison is introduced into the stomach, the latter seems slightly inflamed. Contrary to Brodie, it is maintained, that the Upas poison does not kill by palsying the heart, as in some cases the heart was seen to beat lively even after death.

---

